# Long-Term Surgical Outcomes of Scleral Flap versus Scleral Pocket Technique for Sutureless Intrascleral One-Piece Lens Fixation

**DOI:** 10.3390/jcm13154452

**Published:** 2024-07-29

**Authors:** Paola Marolo, Paolo Caselgrandi, Michele Gaidano, Fabio Conte, Guglielmo Parisi, Enrico Borrelli, Matteo Fallico, Mario Damiano Toro, Luca Ventre, Agostino S. Vaiano, Michele Reibaldi

**Affiliations:** 1 Department of Ophthalmology, University of Turin, 10126 Turin, Italy; 2 Department of Ophthalmology, University of Catania, 95123 Catania, Italy; 3 Department of General Ophthalmology, Medical University of Lublin, 20079 Lublin, Poland; 4 Eye Clinic, Public Health Department, Federico II University, 80131 Naples, Italy; 5 Department of Ophthalmology, Beauregard Hospital, 11100 Aosta, Italy; 6 Institute of Ophthalmology, Santa Croce e Carle Hospital, 12100 Cuneo, Italy

**Keywords:** secondary intraocular lens implantation, sutureless scleral fixation, sutureless intrascleral one-piece lens, scleral flap, scleral pocket, long-term outcomes

## Abstract

**Objectives:** This study compared long-term surgical outcomes of the scleral flap versus scleral pocket technique for sutureless intrascleral one-piece intraocular lens (IOL) fixation. **Methods**: A retrospective comparative study was conducted at a single center, involving consecutive patients undergoing sutureless intrascleral one-piece IOL implantation, between January 2020 and May 2022. Eyes were divided into two groups based on the surgical technique: group 1 underwent scleral flap *(n* = 64), and group 2 received scleral pocket technique (*n* = 59). Visual acuity, refractive outcomes, and complications were assessed over a minimum 24-month follow-up period. **Results**: Both groups showed improvements in best-corrected visual acuity (BCVA), increasing from 0.84 ± 0.56 logMAR at baseline to 0.39 ± 0.23 logMAR (*p* = 0.042) at 24 months in group 1 and from 0.91 ± 0.63 logMAR at baseline to 0.45 ± 0.38 logMAR (*p* = 0.039) at 24 months in group 2. No significant differences in BCVA were observed between the groups at baseline (*p* = 0.991), 12 (*p* = 0.496) and 24 months (*p* = 0.557). Mean spherical equivalent (−0.73 ± 1.32 D in group 1 and −0.92 ± 0.99 D in group 2, *p* = 0.447), refractive prediction error (−0.21 ± 1.1 D in group 1 and −0.35 ± 1.8 D in group 2, *p* = 0.377), and surgically induced astigmatism (0.74 ± 0.89 D in group 1 and 0.85 ± 0.76 in group 2, *p* = 0.651) were comparable between the two groups. An IOL tilt of 5.5 ± 1.8 and 5.8 ± 2.0 degrees (*p* = 0.867) and an IOL decentration of 0.41 ± 0.21 mm and 0.29 ± 0.11 mm (*p* = 0.955) were obtained, respectively, in group 1 and group 2 at 24 months. Mean endothelial cell density remained stable at 24 months in both groups (*p* = 0.832 in group 1 and *p* = 0.443 in group 2), and it was 1747.20 ± 588.03 cells/mm^2^ in group 1 and 1883.71 ± 621.29 cells/mm^2^ in group 2 (*p* = 0.327) at baseline, 1545.36 ± 442.3 cells/mm^2^ in group 1 and 1417.44 ± 623.40 cells/mm^2^ in group 2 (*p* = 0.483) at 24 months. No cases of endophthalmitis were observed. **Conclusions**: The scleral pocket technique for sutureless intrascleral one-piece IOL fixation is comparable to the traditional scleral flap technique in terms of long-term visual outcomes and safety. The scleral pocket technique offers a simplified approach and a viable option even for less experienced surgeons.

## 1. Introduction

Secondary intraocular lens (IOL) implantation in aphakic eyes without capsular support has been addressed over the years with a wide variety of surgical techniques. To date, sutureless scleral fixation techniques are the most commonly used because of the advantages they have shown compared with trans-scleral suturing IOLs and iris claw IOLs [1,2,3,4,5,6]. However, even sutureless techniques for three-piece IOLs present complications, such as IOL tilt, haptic slippage, and IOL dislocation [7,8,9].

In 2015, the FIL-SSF lens (Soleko S.p.A., Rome, Italy) was introduced, specifically designed for sutureless scleral fixation. It is a hydrophilic acrylic single-piece IOL with two anchor haptics designed for intrascleral positioning, allowing for standardized and reproducible surgery. This lens has shown promising results [10], and surgeons have gradually refined the implantation technique to minimize complications, improve refractive outcomes, and make the implantation procedure simpler, more practical, and faster.

In addition to the classic scleral flap technique [11,12,13,14,15,16], in which two quadrangular flaps are partially dissected from the sclera and positioned over the lens haptics, various surgical variants have been proposed over time. These techniques have included the implantation of the sutureless intrascleral one-piece lens alone or in combination with vitrectomy [17,18,19]. The scleral pocket technique has recently been proposed [18,20,21,22]. This involves creating two self-sealing scleral pockets to reduce operative time, minimize iatrogenic damage to the sclera, and prevent friction between the haptics and the conjunctiva. The scleral pocket technique theoretically simplifies the procedure by obviating the need for the surgeon to extensively manipulate the sclera to create two quadrangular flaps that must then be repositioned and secured. This renders the technique more accessible and quicker to perform, potentially broadening its applicability among surgeons with varying levels of expertise. Moreover, recent studies [20,23] suggest that the scleral pocket technique could reduce the risk of conjunctival erosion and infection over time. This is because of the fact that the pocket, contained within the scleral thickness and opened externally by a single incision, may theoretically better contain the IOL haptics compared to the flap, which is anchored to the sclera on only one of its sides and is subject to potential displacements [14]. However, it remains uncertain whether a technique that does not involve placing a flap over the emerging haptic could result in IOL tilting, decentration, potential long-term refractive errors, and potential complications due to exposure of the IOL haptics. To date, only short-term results of the scleral pocket technique have been reported, and no studies providing a long-term comparison with the traditional flap technique are available.

The purpose of our study was to evaluate the long-term surgical outcomes of the scleral flap technique compared with the scleral pocket technique to determine whether the more recent scleral pocket technique constitutes a viable surgical option. Specifically, we evaluated visual acuity, refractive outcomes, and complication rates over a minimum of 24 months.

## 2. Materials and Methods

We performed a single-center, retrospective comparative study on consecutive eyes undergoing sutureless intrascleral one-piece IOL implantation between January 2020 and May 2022. The procedures were performed by three experienced vitreoretinal surgeons (M.R., L.V., and G.P.) at “Città della Salute e della Scienza” Hospital, University of Turin, Italy. The study protocol complied with the tenets of the Declaration of Helsinki and was reviewed and approved by the institutional ethics committee. All patients signed a written informed consent, agreeing to participate.

Only eyes that were diagnosed with aphakia or IOL/lens luxation/subluxation and with a follow-up of at least 24 months were included in the study. To ensure the homogeneity of the study population, eyes with the following clinical situations were excluded: (1) high refractive errors (myopia > 6 diopters, hypermetropia, or astigmatism > 3 diopters); (2) prior open globe trauma; (3) prior corneal surgery or buckling surgery; (4) amblyopia; (5) ocular comorbidities that may affect best-corrected visual acuity (BCVA), including macular disorders such as age-related macular degeneration and optic nerve diseases such as glaucoma; (6) sclero-corneal tunnel enlargement and need of sclero-corneal sutures at the time of surgery; and (7) intraoperative complications such as haptic rupture.

According to the fixation technique adopted by the surgeon, eyes were divided into two groups: group 1, implanted with the scleral flap technique (from January 2020 to November 2021), and group 2, implanted with the scleral pocket technique (from December 2021 to May 2022). Type of vitrectomy (23 or 25 Gauge) and duration of surgery were also recorded. To minimize inter-surgeon variability, all surgeons followed a standardized protocol for both techniques.

### 2.1. Examination

A complete ophthalmologic examination was performed prior to treatment and at 12 months and 24 months after surgery. Demographics and clinical characteristics including gender, age, axial length, preoperative diagnosis, and intraocular pressure were recorded.

BCVA, subjective refraction, spherical equivalent (SE), refractive prediction error, surgically induced astigmatism, as well as IOL decentration and tilt measurement were evaluated. Decimal BCVA was converted to logarithm of the minimal angle of resolution (logMAR). Biometric parameters were obtained with the IOLMaster 700 (Carl Zeiss Meditec Inc., Dublin, CA, USA) prior to pupil dilation. Biometry was feasible in all eyes. Anterior chamber depth (ACD) independent formulae were used for IOL power calculation. The refractive prediction error (PE) was calculated as the difference between postoperative objective refraction expressed as SE and the predicted SE of the refraction obtained from preoperative biometry using the SRKT formula. A negative PE indicated a myopic result whereas a positive PE indicated a hyperopic result as compared with the expected refraction. Decentration and tilt were measured using the inbuilt software of the Casia 2 AS-OCT SS-1000 (Tomey Corporation, Nagoya, Aichi, Japan), analyzing and modelling the IOL position over 360 degrees.

Intraoperative and postoperative complications were recorded. The endothelial cell count was obtained using the EM-3000 (Tomey Corporation, Nagoya, Aichi, Japan) noncontact specular microscope.

The main objective of this study was to compare surgical outcomes at 12 and 24 months of the scleral flap versus scleral pocket technique for sutureless intrascleral one-piece lens fixation. Specifically, the study aims to evaluate visual acuity, refractive outcomes, and complication rates over a minimum of 24 months.

### 2.2. Surgical Technique

All cases were performed by three experienced vitreoretinal surgeons (M.R., L.V., and G.P.) at “Città della Salute e della Scienza” Hospital, University of Turin, Italy with the patient under peribulbar block. To reduce variability between surgeons, all surgeons adhered to a uniform protocol for both techniques.

**Figure 1:**
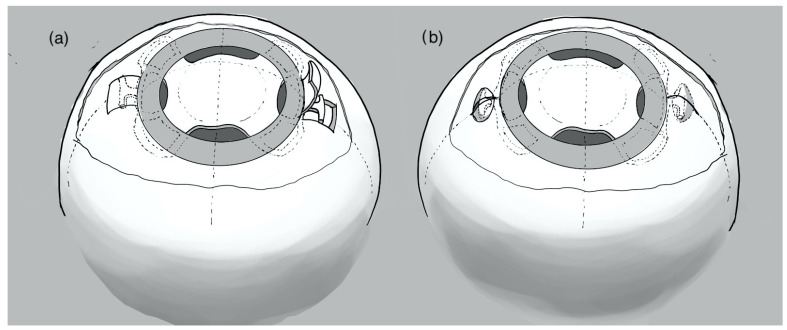
Schematic representation of the two surgical techniques: scleral flap (**a**) versus slceral pocket (**b**). The figures show the sutureless intrascleral one-piece lens in the background, indicating its placement in the posterior chamber with sutureless scleral fixation of the haptics. (**a**) illustrates the scleral flap technique: on the right, the scleral flap before closure on the IOL haptics, and on the left, the flap closed. (**b**) shows the scleral pocket technique: on the left, the scleral pocket still open with the IOL haptics inside, and on the right, the pocket closed.

In the scleral flap technique (group 1), a conjunctival peritomy was performed nasally and temporally. Then, two anteriorly hinged partial-thickness 4 × 4 mm scleral flaps were created 180° apart along the horizontal meridian, and two opposite sclerotomies were performed under each flap at 2 mm from the limbus with a 25-gauge needle within the scleral bed. The scleral flap was then overlapped to the underlying sclera and closed with fibrin glue, and also the conjunctiva was closed with fibrin glue above the scleral flap. The scleral pocket technique (group 2) was previously described [20]. After a conjunctival peritomy was performed nasally and temporally, two straight incisions running posteriorly to the limbus for 2.5 mm were made with a crescent blade. The sclera was dissected to create two opposite pockets at each side of the incisions, and a sclerotomy was performed at 1.75 mm from the limbus within each incision with a 25-gauge needle. Scleral incisions were sutured with a butterfly or cross-stitch [20], and the knot was then placed inside the scleral incision and the conjunctiva was closed with fibrin glue. A schematic representation of the two techniques has been provided in Figure 1.

Afterwards, in both groups, a clear corneal tunnel of 2.2 mm and a side port were created along the axis of the scleral flap, and the IOL was slowly injected into the anterior chamber (AC). Once fully opened, the leading plug of the haptic was grabbed and externalized with a 25-gauge crocodile-tip forceps inserted through the sclerotomy. Finally, a second forceps was passed through the side port to grab the trailing plug that was transferred to the first forceps to be externalized entering the opposite sclerotomy.

In all surgeries, a 25–23-gauge pars plana vitrectomy (PPV) was performed, using the Constellation Vision System (Alcon Laboratories, Fort Worth, TX, USA), and trocars were inserted avoiding 0° and 180° axes. A 25-gauge PPV was preferred in subluxated–luxated IOL or aphakia cases, and a 23-gauge PPV in the case of a dropped lens extraction. In case of any dropped fragments from the nucleus, they were removed with a vitrectome probe or a fragmatone. For subluxated–luxated IOL, it was released from capsular-lens remnants, cut into 2 pieces, and extruded from the sclero-corneal tunnel for the sutureless intrascleral one-piece IOL implantation enlarged up to 3.0 mm.

### 2.3. Statistical Analysis

Snellen visual acuity was obtained and converted into the logarithm of the minimum angle of resolution (logMAR) for statistical analysis. Mean and standard deviation (SD) were computed for continuous variables, while frequency and percentage were calculated for qualitative variables. The normal distribution of continuous variables was evaluated by using the Shapiro–Wilk test. Differences between the two groups were explored with the Mann–Whitney U test and the Student’s *t*-test for parametric and non-parametric variables, respectively. Categorical variables were tested using the chi-square test or Fisher’s exact test when necessary. The average visual change in each group was calculated during follow-up. In each group, mean visual acuity values recorded at different time points were compared using the ANOVA test. Statistical analysis was performed using the Statistical Package for Social Sciences (28.0.1.0 version IBM SPSS Statistic Inc., Chicago, IL, USA). A *p* value ≤ 0.05 was considered significant.

## 3. Results

A total of 123 eyes (123 patients) were enrolled. Group 1 (scleral flap) included 64 eyes from 64 patients from 68 to 84 years old (mean age: 75.1 ± 8.1 years), consisting of 30 women and 34 men, with a mean follow-up of 27.6 ± 3.3 months; group 2 (scleral pocket) included 59 eyes from 59 patients from 65 to 82 years old (mean age: 73.3 ± 7.8 years), consisting of 29 women and 30 men, with a mean follow-up of 28.8 ± 4.6 months. Preoperative diagnoses and clinical characteristics at baseline are summarized in Table 1. Baseline characteristics such as gender, age, intraocular pressure, and axial length were comparable between the two groups, minimizing selection bias (*p* > 0.05). In group 1, 29.7% of eyes were diagnosed with aphakia, 43.8% of eyes presented a dislocated IOL, and 26.6% of eyes had a dislocated lens; in group 2, 20% of eyes were diagnosed with aphakia, 24% of eyes presented a dislocated IOL, and 15% of eyes had a dislocated lens. In both groups, a 25-Gauge PPV was preferred to a 23-Gauge PPV (74.4% eyes in group 1 and 74.6% of eyes in group 2). The duration of surgery was not different between the two groups, with a mean duration of 62.5 ± 15.3 min in group 1 and 60.1 ± 16.7 min in group 2 (*p* = 0.725). The mean follow-up was 27.6 ± 3.3 months in group 1 and 28.8 ± 4.6 months in group 2 (*p* = 0.231).

### 3.1. Visual and Refractive Outcomes

**Figure 2:**
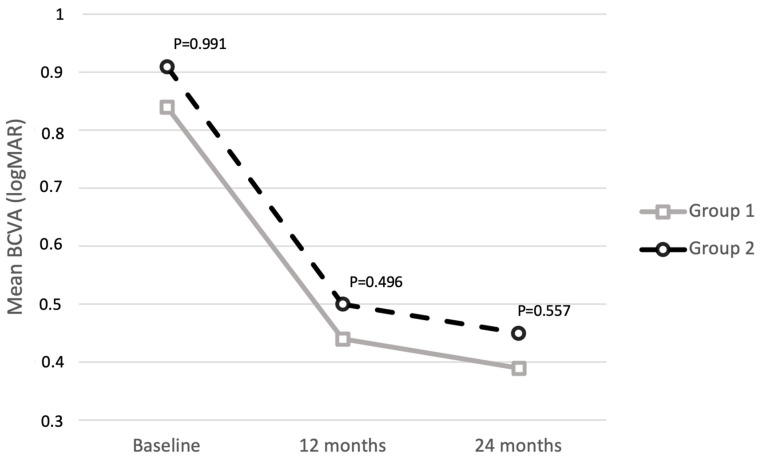
Mean best-corrected visual acuity (BCVA) in the two study groups throughout the follow-up.

Mean BCVA significantly improved throughout the follow-up period in both group 1 and group 2, increasing from 0.84 ± 0.56 logMAR at baseline to 0.44 ± 0.35 logMAR at 12 months (*p* = 0.003) to 0.39 ± 0.23 logMAR at 24 months (*p* = 0.042) in group 1, and from 0.91 ± 0.63 logMAR at baseline to 0.50 ± 0.44 logMAR at 12 months (*p* = 0.005) to 0.45 ± 0.38 logMAR at 24 months (*p* = 0.039) in group 2. No significant differences were observed between the two groups at baseline (*p* = 0.991), 12 months (*p* = 0.496), and 24 months (*p* = 0.557). Figure 2 shows the improvement in BCVA over 24 months for both groups, indicating no significant difference in visual outcomes. Table 2 shows refractive outcomes at the different time points in the two groups.

**Figure 3:**
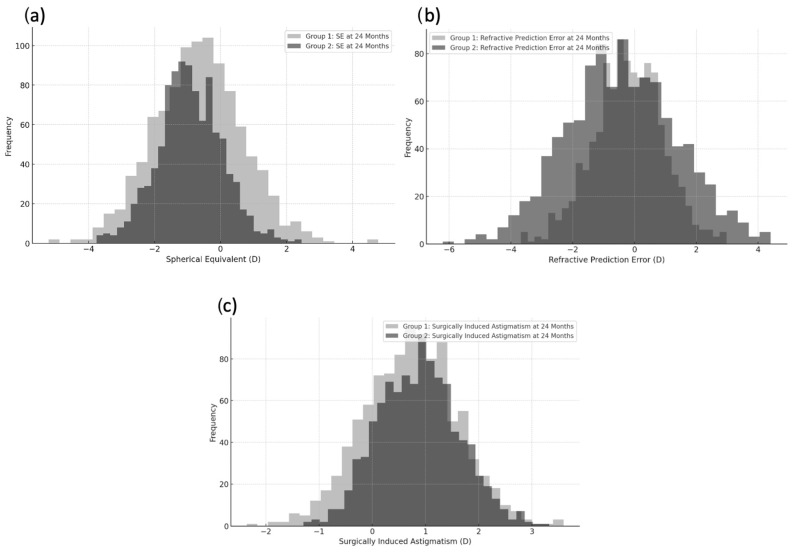
Frequency distribution of spherical equivalent (**a**), refractive prediction error (**b**), and surgically induced astigmatism (**c**) in the two study groups.

The mean SE significantly changed from 12.4 ± 7.78 D at baseline to −0.73 ± 1.32 D at 24 months in group 1 (*p* < 0.001) and from 13.2 ± 6.45 D at baseline to −0.92 ± 0.99 D at 24 months in group 2 (*p* < 0.001). No significant differences were observed between the two groups at baseline (*p* = 0.679), 12 months (*p* = 0.275), and 24 months (*p* = 0.447). The mean refractive PE and SIA were not different between group 1 and group 2 (*p* = 0.377 and *p* = 0.651, respectively). Figure 3 displays the frequency distribution of the data of SE, refractive PE, and SIA at 24 months for each group, giving a visual representation of the variability and central tendency of the three refractive data mentioned above in the two study groups.

Moreover, IOL tilt and decentration were comparable between the two groups at the different time points (*p* = 0.744 at 12 months and *p* = 0.867 at 24 months for IOL tilt, *p* = 0.879 at 12 months and *p* = 0.955 at 24 months for decentration).

**Figure 4:**
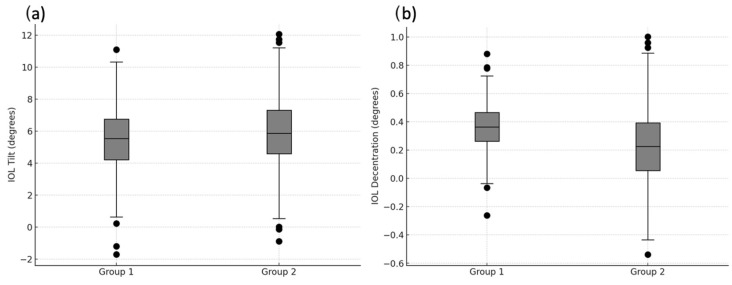
Boxplot of IOL tilt (**a**) and decentration (**b**) at 24 months in the two study groups.

Figure 4 shows the boxplot for IOL tilt and decentration at 24 months for each group, illustrating the variability and central tendency as well as highlighting how the lens might tilt or shift postoperatively.

### 3.2. Endothelial Cell Density, Complications, and Reintervention

**Figure 5:**
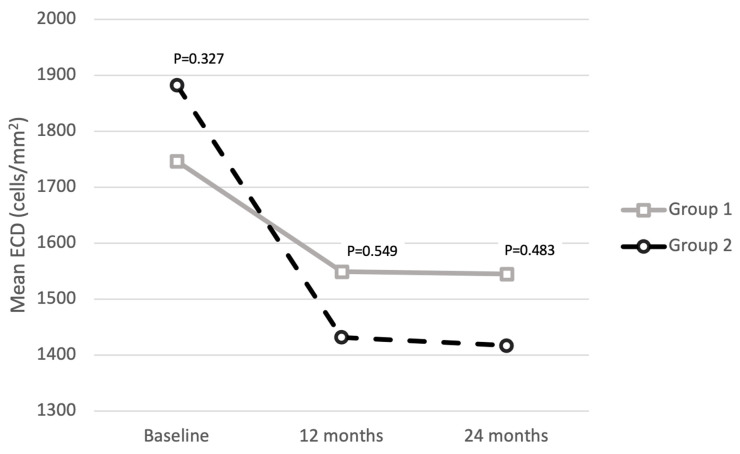
Mean endothelial cell density in the two study groups throughout the follow-up.

Mean endothelial cell density (ECD) did not undergo statistically significant changes at 12 and 24 months within the two groups (*p* = 0.932 at 12 months and *p* = 0.832 at 24 months in group 1, *p* = 0.764 at 12 months and *p* = 0.443 at 24 months in group 2). Mean ECD was 1747.20 ± 588.03 cells/mm^2^ in group 1 and 1883.71 ± 621.29 cells/mm^2^ in group 2 at baseline (*p* = 0.327), 1549.94 ± 657.44 cells/mm^2^ in group 1 and 1432.33 ± 659.43 cells/mm^2^ in group 2 at 12 months (*p* = 0.549), and 1545.36 ± 442.3 cells/mm^2^ in group 1 and 1417.44 ± 623.40 cells/mm^2^ in group 2 at 24 months (*p* = 0.483). Figure 5 shows the stability in ECD over 24 months for both groups, indicating no significant difference in outcomes concerning corneal safety.

Postoperative complications and reintervention are reported in Table 3. There was no difference in these terms between the two surgical techniques. In group 1, two patients developed persistent corneal edema at 1 month and underwent corneal endothelial transplantation (Descemet Stripping Endothelial Keratoplasty, DMEK). Three patients required a revision vitrectomy, one of whom experienced a retinal detachment occurring at 6 months, while the remaining two patients had a massive vitreous hemorrhage that did not resolve spontaneously. One patient showed persistent postoperative hypotony at 1 month and required the placement of additional sutures at the scleral flap. In group 2, one patient developed corneal edema that did not respond to corticosteroid therapy and underwent DMEK. Two patients had a massive vitreous hemorrhage that did not resolve spontaneously and required revision vitrectomy. In three cases, an IOL haptic dislocated outside the scleral pocket, causing conjunctival erosion in one case, which required conjunctival suturing initially and a scleral patch in a second reoperation. No cases of endophthalmitis were observed.

## 4. Discussion

In this study, we investigated the long-term surgical outcomes of two different sutureless intrascleral one-piece lens fixation techniques, the traditional scleral flap technique and the newer scleral pocket technique. We obtained comparable visual and refractive outcomes between the two surgical procedures at 24 months, as well as a similar rate of long-term complications.

The Soleko FIL-SSF single-piece foldable acrylic lens was introduced for sutureless intrascleral fixation, having been specifically designed for this purpose. Previous reports documented the advantages of implanting this IOL in the absence of capsular support, including the absence of haptic manipulation and its self-centration thanks to its single-piece design [10]. Initial studies show good visual outcomes and a low rate of short-term complications [11,13,14]. In a prospective observational case series of 32 eyes, Barca et al. [11] found significant visual improvement at 8 months of follow-up, with no significant lens tilting and minor complications. These favorable surgical and functional results were confirmed in case series with a larger sample size [12,13,14].

Such studies used the classical scleral flap technique for implantation, in which two anteriorly hinged partial-thickness scleral flaps measuring from 3 to 4 mm were created and then closed over the two T haptics emerging from the sclera with sutures or fibrin glue. The creation of the flap theoretically provides greater security regarding the maintenance of the correct positioning of the lens over time and protection from potential infectious agents. However, complications such as conjunctival erosion and plug externalization have been reported [14]. This could lead to long-term infectious complications. Additionally, the creation of a partial-thickness scleral flap is a challenging surgical procedure that requires the surgeon to have considerable experience and familiarity with the technique.

Surgeons have refined the technique to make the IOL fixation procedure more practical, widely accessible, and capable of achieving the same positive long-term surgical outcomes with less manipulation of ocular tissue. Consequently, the scleral pocket technique was introduced as a simplified method of intrascleral fixation, which secures the T-shaped IOL haptics within the scleral wall without requiring scleral flaps. Gabai et al. [22], in a case series of 13 patients who underwent lens implantation using the scleral pocket technique, reported an improvement in mean corrected distance visual acuity from 0.75 ± 0.5 logMAR to 0.28 ± 0.3 logMAR, with complications occurring rarely over a 7-month period. Other studies showed similar results at 6 and 12 months [20,24]. In a larger prospective interventional study involving 60 eyes that underwent the procedure with two opposite self-sealing scleral pockets combined with vitrectomy, the authors confirmed these results with a short-term follow-up of 4 months [18].

However, as a relatively new technique, there are no long-term results to confirm that this method is interchangeable with the classic one. Additionally, complications such as vitreous hemorrhage, haptic exposure, and endophthalmitis have been reported [10,22]. In the literature, the only comparison between the classic technique and the scleral pocket was published by Fiore et al. [23] in a retrospective comparative study of 23 eyes with scleral pocket versus nine eyes with scleral flap, with an average follow-up of about 4 months. In this study, the lens was successfully managed without any significant differences between the two surgical techniques. To our knowledge, no larger or longer-term comparative studies are available.

Regarding visual outcomes, our study observed an improvement in visual acuity in both groups during the follow-up period, with no significant differences between the two groups at 12 and 24 months. Additionally, the refractive outcomes were also positive. In an era of increasing refractive expectations by patient, the importance of refractive outcomes becomes more evident, especially for patients regaining good visual acuity following surgery. On this topic, little has been published to date. Recently, in a prospective randomized study comparing three-piece and one-piece intrascleral fixated lenses, refractive outcomes and lens power calculation were assessed, showing similar refractive predictability in both groups and a better IOL tilt of 6.45 ± 2.03° in the one-piece intrascleral sutureless implanted eyes. Surgically induced astigmatism was evaluated by the same group at 24 weeks, showing a predictable value of <1D in a group of 27 eyes. In our study, the results were consistent with those reported and demonstrated that the two techniques were comparable. Importantly, at 24 months, no significant IOL decentration was detected.

No statistically significant differences were noted in the complications reported between the two groups. Endothelial cell density did not undergo significant reductions over the two years in either group, indicating safety of the technique concerning the corneal endothelium. Long-term corneal decompensation occurred in two cases in group 1 and one case in group 2, which were resolved with a corneal endothelial transplant. It should be noted that, at 24 months, no cases of endophthalmitis were detected in either group. In the group 2, scleral pocket, there was IOL haptic exposure in three cases, with only one case resulting in conjunctival erosion, which was resolved with the application of a scleral patch.

Concerning the timing of surgery, no significant reduction in duration was observed for group 2. This was likely due to the surgeon’s choice not to suture the flap in group 1 (using glue instead) but to place sutures on the pocket in group 2. If the surgeon had used glue, the times would probably have been shorter.

Our study has some limitations that should be considered when interpreting the results. First is the retrospective nature of the study, which could lead to potential biases such as selection bias, recall bias, information bias, and confounding bias. These biases arise because data collection was not controlled prospectively, and thus, the allocation of subjects to different treatment arms was not randomized. To mitigate such biases, future studies should ideally be prospective and randomized to ensure more controlled and reliable comparisons between treatment groups. Furthermore, surgery was performed by three different surgeons; however, the three surgeons worked in the same center and employed a shared standardized surgical technique for both procedures. Moreover, increasing the sample size would enhance the statistical power of the study, reducing the risk of Type II errors and increasing the reliability of the findings. This would be particularly important in detecting small but clinically significant effects. Lastly, standardizing data collection methods could further enhance the accuracy and generalizability of the results. These steps will help to address the biases inherent in retrospective studies and strengthen future research outcomes.

Findings suggesting the scleral pocket technique as a simpler and equally effective alternative to the scleral flap technique have significant implications for clinical practice, especially for less experienced surgeons. This research indicates that the scleral pocket technique offers several practical advantages. Firstly, it involves a less complex procedure with a smaller incision and potentially shorter surgical time. This could result in a less steep learning curve for novice surgeons, allowing them to acquire surgical skills more quickly and safely. Moreover, our study has shown that outcomes with the scleral pocket technique are comparable to those with the traditional scleral flap technique in terms of long-term success rates and complications. This equivalence provides significant reassurance to surgeons that opting for the simpler technique does not compromise treatment effectiveness and safety. Adopting the scleral pocket technique could therefore be a prudent strategy to enhance the safety and precision of surgical procedures. Starting with a less complicated technique may reduce the risk of complications and improve the overall patient experience during and after surgery.

## 5. Conclusions

In conclusion, this study shows that the recent implantation technique of the sutureless scleral fixation one-piece IOL using a scleral pocket is comparable to the traditional method involving a scleral flap. Our findings indicate good refractive outcomes and long-term safety for both techniques. This suggests that the more recent and simpler scleral pocket technique is not only feasible but also a viable option, particularly beneficial for surgeons who may have less experience with the implantation procedure.

For future research, there is a critical need to conduct studies with larger sample sizes and extended follow-up periods to further validate these findings. Establishing standardized training protocols and comparing outcomes across different patient demographics would provide additional insights into optimizing surgical techniques and ensuring consistent, high-quality care in this surgery. By focusing on these areas, we can advance towards enhancing surgical outcomes and expanding accessibility to effective techniques such as the scleral pocket method.

## Figures and Tables

**Table 1:** Demographics and clinical characteristics of the two study groups at baseline.

	Group 1 Scleral Flap *n* = 64	Group 2 Scleral Pocket *n* = 59	*p* Value
Women, *n* (%)	30 (46.9)	29 (49.2)	0.801
Age (years), mean (±SD)	75.1 (8.1)	73.3 (7.8)	0.525
Axial length (mm), mean (±SD)	24.1(1.9)	23.9 (1.6)	0.777
Preoperative diagnosis, *n* (%)			
Aphakia	19 (29.7)	20 (33.9)	
Dislocated IOL	28 (43.8)	24 (40.7)	
Dislocated lens	17 (26.6)	15 (25.4)	
IOP (mm Hg), mean (±SD)	17.5 (3.3)	19.1 (2.6)	0.711
Features of the surgery			
Type of vitrectomy, *n* (%)			0.886
23-Gauge	17 (26.6)	15 (25.4)	
25-Gauge	47 (73.4)	44 (74.6)	
Duration of surgery (minutes), mean (±SD)	62.5 (15.3)	60.1 (16.7)	0.725
Follow-up (months), mean (±SD)	27.6 (3.3)	28.8 (4.6)	0.231

SD: standard deviation; IOL: intraocular lens; IOP: intraocular pressure.

**Table 2:** Refractive outcomes in the two study groups.

	Group 1 Scleral Flap *n* = 64	Group 2 Scleral Pocket *n* = 59	*p* Value
SE (D) mean (±SD)			
Baseline	12.4 (7.78)	13.2 (6.45)	0.679
12 months	−0.70 (1.57)	−0.96 (1.08)	0.275
24 months	−0.73 (1.32)	−0.92 (0.99)	0.477
Refractive PE (D), mean (±SD)	−0.21 (1.1)	−0.35 (1.8)	0.377
SIA (D), mean (±SD)	0.74 (0.89)	0.85 (0.76)	0.651
IOL tilt (degree), mean (±SD)			
12 months	5.6 (3.6)	6.2 (2.2)	0.744
24 months	5.5 (1.8)	5.8 (2.0)	0.867
Decentration (mm), mean (±SD)			
12 months	0.36 (0.15)	0.22 (0.25)	0.879
24 months	0.41 (0.21)	0.29 (0.11)	0.955

SE: spherical equivalent; SD: standard deviation; PE: prediction error; SIA: surgically induced astigmatism; IOL: intraocular lens.

**Table 3:** Complications and reinterventions.

	Group 1 Scleral Flap (*n* = 64)	Group 2 Scleral Pocket (*n* = 59)	*p* Value
Postoperative complications, *n* (%)			
Cystoid macular edema	3 (4.69)	2 (3.39)	0.716
Hyphema	3 (4.69)	4 (6.78)	0.617
Vitreal hemorrhage	4 (6.25)	3 (5.08)	0.780
Hypotony	5 (7.81)	4 (6.78)	0.826
IOP spike	2 (3.13)	2 (3.39)	0.934
Persistent corneal edema	2 (3.13)	1 (1.69)	0.607
IOL haptic exposure	0 (0)	3 (5.08)	-
Conjunctival erosion	0 (0)	1 (1.69)	-
Retinal detachment	1 (1.56)	0 (0)	-
Endophthalmitis	0 (0)	0 (0)	-
Reintervention, *n* (%)			
Endothelial corneal transplant	2 (3.13)	1 (1.69)	0.607
Vitrectomy	3 (4.69)	2 (3.39)	0.947
Scleral flap or scleral pocket sutures	1 (1.56)	0 (0)	-
Conjunctival sutures	0 (0)	1 (1.69)	-
Scleral patch	0 (0)	1 (1.69)	-

IOP: intraocular pressure; IOL: intraocular lens.

## Data Availability

The data that support the findings of this study are available from the corresponding author upon reasonable request.
